# Polyphasic Characterisation of *Cedecea colo* sp. nov., a New Enteric Bacterium Isolated from the Koala Hindgut

**DOI:** 10.3390/microorganisms8020309

**Published:** 2020-02-24

**Authors:** Jarryd M. Boath, Sudip Dakhal, Thi Thu Hao Van, Robert J. Moore, Chaitali Dekiwadia, Ian G. Macreadie

**Affiliations:** 1 School of Science, RMIT University, Bundoora, Victoria 3083, Australia; jarrydboath@gmail.com (J.M.B.); sudip.dhakal@rmit.edu.au (S.D.); thithuhao.van@rmit.edu.au (T.T.H.V.); rob.moore@rmit.edu.au (R.J.M.); 2 Australian Microscopy & Microanalysis Research Facility, RMIT University, Melbourne, Victoria 3000, Australia; Chaitali.dekiwadia@rmit.edu.au

**Keywords:** koala microbiome, polyphasic taxonomy, extremophiles, novel species

## Abstract

The *Cedecea* genus is comprised of six rarely isolated species within the *Enterobacteriaceae* family. Representatives are Gram-negative motile bacilli, and are typically oxidase-negative, lipase-positive and resistant to colistin and cephalothin. In this study, a putative novel *Cedecea* species (designated strain ZA_0188^T^), isolated from the koala hindgut, was characterised using a polyphasic taxonomic approach. Maximum average nucleotide identity (ANI) and 16S ribosomal RNA (rRNA) similarity scores well below thresholds of species demarcation were reported, at 81.1% and 97.9%, respectively. Multilocus phylogenetic analysis indicated strain ZA_0188^T^ was most similar to but divergent from recognised *Cedecea* species. The isolate’s genomic G+C content was determined as 53.0 mol%, >1% lower than previously reported in *Cedecea*. Phenotypically, strain ZA_0188^T^ was distinct from recognised *Cedecea* species such as colistin- and cephalothin-sensitive, lipase-, sorbitol-, sucrose-, and Voges-Proskauer-negative, and melibiose-, arabinose-, arginine-, and rhamnose-positive. In preliminary experiments, strain ZA_0188^T^ exhibited cellulase activity and high-level tolerance to eucalyptus oil compared to other enteric species surveyed. Collectively, these findings suggest that strain ZA_0188^T^ represents a novel enteric species, for which the name *Cedecea colo* is proposed.

## 1. Introduction

*Eucalyptus* species, which dominate the Australian landscape, provide an abundant food source for arboreal animals, including koalas (*Phascolarctos cinereus*), ringtail possums (*Pseudocheirus peregrinus*), and greater gliders (*Petauroides volans*) [1]. Compared to other plant genera, eucalyptus foliage lacks nutritional value, consisting primarily of recalcitrant structural polymers such as cellulose and lignin [2]. Eucalypt polyphenols such as tannins bind proteins in the folivore gut to form tannin-protein complexes, blocking assimilation of valuable substrates [3]. Moreover, eucalyptus plant secondary metabolites (PSMs), which included flavonoids, terpenes and formylated phloroglucinols, are powerful antimicrobials and are highly toxic and immunomodulating in animals [4]. 

In response to natural varying PSM concentrations, folivore species reduce their overall food intake, thereby regulating ingestion of harmful PSMs and avoiding intoxication [5,6,7]. Notwithstanding, animals subsisting entirely on eucalyptus foliage, such as koalas, rely on their native gut microbes for detoxification and the degradation of leaf components [8,9]. Microorganisms able to colonise such inhospitable environments may also withstand the extreme physicochemical conditions central to many industrial processes and thus are of significant interest to biotechnologists. Similarly, species capable of degrading recalcitrant compounds may host novel enzymatic pathways yet to be exploited by industry. 

The *Enterobacteriaceae* family is composed Gram-negative bacillus-shaped bacteria, which are generally oxidase-negative, motile, nonspore-forming, and able to reduce nitrate [10]. *Enterobacteriaceae* species such as *Escherichia coli*, *Salmonella typhimurium* and *Klebsiella pneumoniae* have been linked with several human disease states [10]. Representatives of this family have been isolated from a wide range of sources, though are common to soil, water and the gastrointestinal tract of warm-blooded animals. Several members of this group, including *Serratia marcescens*, have previously been reported in koala microbiome studies [11,12]. 

The *Cedecea* genus is comprised of six rarely isolated species within the *Enterobacteriaceae* family. Representatives are Gram-negative, motile bacilli and are typically oxidase-negative, lipase-positive and resistant to colistin and cephalothin. The aim of this study was to perform comprehensive characterisation of a putative novel *Cedecea* species, designated strain ZA_0188^T^, isolated from the koala hindgut. A polyphasic taxonomic approach was employed and the results support the assertion that strain ZA-0188^T^ represents a new member of the *Cedecea* genus. To our knowledge, this is also the first time a *Cedecea* species has been reported in a koala faecal sample.

## 2. Materials and Methods 

### 2.1. Isolation and Cell Culture

Strain ZA-0188^T^ was recovered from a fresh scat sample collected from the Phillip Island Koala Conservation Centre (Phillip Island Nature Parks, Victoria, Australia) and stored as described by Wedrowicz et al. [13]. Scats were ground up, serially diluted in 0.8% NaCl, plated onto nutrient agar (NA), and incubated at 37 °C in aerobic conditions. After 2 days, a single colony was streak diluted from the mixed culture plate to a fresh NA plate to obtain a pure culture for subsequent analysis. The strain was grown routinely on plates containing Luria-Bertani (LB) medium agar in aerobic conditions at 37 °C. The microorganism has been isolated on multiple occasions, from multiple koalas, and has also been isolated from koalas at Kyabram Fauna Park, Victoria, Australia. 

### 2.2. Single and Multilocus Phylogenetic Sequence Analysis 

The strain ZA-0188^T^ draft genome sequence was generated using an Illumina A5-miseq sequencer. Single-locus analyses of strain ZA-0188’s 16S ribosomal RNA (rRNA) and bacterial heat-shock protein (GroEL) sequences were performed using the megaBLAST algorithm, generating sequence similarity scores to related species present in the National Center for Biotechnology Information (NCBI) database. Average Nucleotide Identity (ANI) two-way scores were generated via an online calculator [14]. Multilocus sequence analysis (MLSA) was performed using the PhyloSift software pipeline, which is freely available online [15]. A dendrogram was constructed using the neighbour-joining algorithm. Dendrogram robustness was assessed by bootstrap analysis from 1000 iterations. 

### 2.3. Morphological and Biochemical and Nutritional Characterisation 

Cell morphology was examined by Scanning Electron Microscopy (SEM), as described by Fischer et al. (2012) [16]. Cells were grown on NA for 72 h at 37 °C prior to microscopy analysis. 

The biochemical and nutritional characteristics of strain ZA-0188^T^ were determined using the API20E system (BioMerieux, Norwest, NSW, Australia) as described by Varettas et al. (1995) [17]. The API20E kit tests for *o*-nitrophenyl-β-d-galactopyranoside (ONPG) hydrolysis; decarboxylation of arginine, lysine and ornithine; citrate utilization; hydrogen sulfide (H_2_S) production; urease, tryptophanase and gelatinase activity; glucose fermentation via the butylene glycol pathway; and fermentation of glucose, mannose, inositol, sorbitol, rhamnose, sucrose, melibiose, amygdalin and arabinose. API20E tests were performed in triplicate and in accordance with the manufacturer’s guidelines. In accordance with the method described by Cowan et al. (1965) [18], catalase and nitrate reductase activity were determined by adding 3% (*w*/*v*) hydrogen peroxide and 7.5% (*w*/*v*) total salt to the growth medium, respectively. Tetramethyl-*p*-phenylenediamine (1%) was used to probe oxidase activity according to the method described by Kovacs et al. (1956) [19]. 

### 2.4. MALDI-TOF Mass Spectroscopy Analysis 

Cells for matrix-assisted laser desorption ionization time-of-flight (MALDI-TOF) mass spectroscopy (MS) analysis were grown overnight at 37 °C under aerobic conditions. Following incubation, a single colony was suspended in 100 µL HPLC-grade water in an Eppendorf tube. The suspension was mixed thoroughly, and cells fixed by adding 900 µL of absolute alcohol. The suspension was then centrifuged at 15,000 RPM for 2 min, supernatant discarded, cell pellets air dried, resuspended in 25 µL of 70% formic acid, and mixed with 100% acetonitrile. This suspension was centrifuged at 15,000 RPM for 2 min, and 1 µL of the suspension was loaded into a 96 well anchor chip plate for MALDI-TOF analysis using a Bruker Biotyper (Preston, Vic., Australia). The spot was air dried before 1 µL HCCA matrix solution was added to the well. The Biotyper instrument was calibrated using appropriate BTS standards, and the cell extract protein content analysed. Using Compass RTC and Flex Control software, identification of strain ZA-0188^T^ was performed by comparing peptide mass fingerprint data with those present in the Bruker database. 

## 3. Results and Discussion

### 3.1. Isolation of strain ZA_0188^T^

Strain ZA-0188 T comprised approximately 1% of the culturable microbes from koala scat samples obtained from the Phillip Island Koala Conservation Centre. It was chosen because of its pink colour. The same microbe has subsequently been isolated from different koalas at Phillip Island Koala Conservation Centre and the Kyabram Fauna Park, suggesting that it may be an intrinsic member of koala hindgut microbiota.

### 3.2. Single-Locus Sequence Analysis of 16S rRNA and GroEL

Single-locus analysis of 16S rRNA gene sequences revealed strain ZA_0188^T^ was most closely related to *Escherichia xiangfangensis* (LMG 27195), returning a sequence similarity score of 97.93% (Table 1). Kim et al. (2014) [20] sought coherence between current and classical methods for differentiating two species. The authors proposed a 16S rRNA gene sequence similarity of 98.65%, as opposed to 70% DNA-DNA hybridisation, considered the gold standard threshold for species demarcation. Due to problems of resolution at the species and subspecies level, the use of 16S rRNA sequence similarity scores alone in bacterial taxonomy is contentious [21]. The *groEL* gene sequence has higher levels of interspecies variation than 16S rRNA, improving resolving power [22]. For this reason, *groEL* sequences are commonly used to generate high-resolution phylogenetic trees, though an equivalent threshold for species demarcation has not been proposed [23]. To further investigate the phylogeny of strain ZA_0188^T^, *groEL* sequences of all closely related species were aligned, returning similarity scores ≤ 92.17% (Table 1).

### 3.3. Multilocus Sequence Analysis 

**Figure 1:**
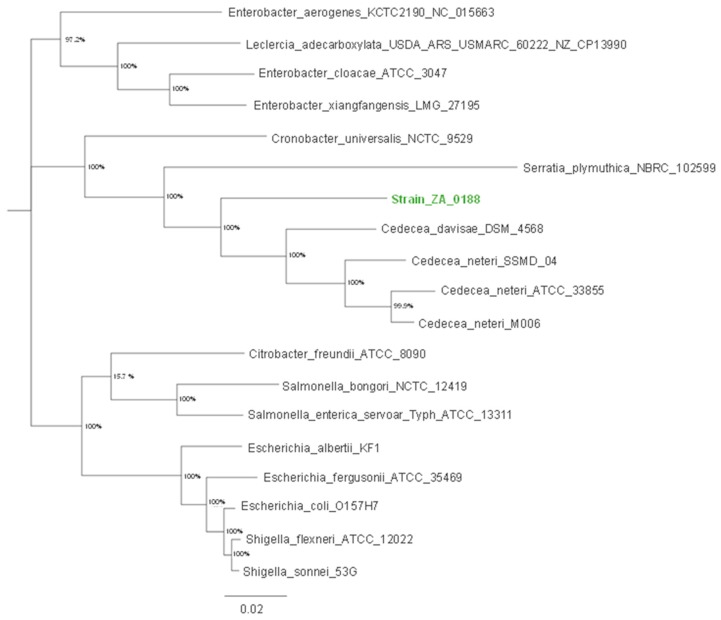
Dendrogram generated using the PhyloSift software pipeline by the neighbour-joining method. Strain ZA_0188^T^ forms a robust clade with Cedecea and Serratia species with 100% bootstrap support. Bootstrap values are shown at each node and were determined from 1000 repetitions. Scale bar is equal to 0.02 substitutions/nucleotides position.

In order to increase resolution, MultiLocus Sequence Analysis (MLSA) comparing strain ZA_0188^T^ and 18 closely related species was performed using the PhyloSift software pipeline. PhyloSift locates, aligns and outputs sequence similarity scores for 42 genetic markers simultaneously and combines the output to infer phylogenetic relationships. The MLSA dendrogram suggested strain ZA_0188^T^ is most closely related to *Cedecea* species, though it forms its own robust clade with 100% bootstrap support, corroborating our assertion that the isolate is a new species within this genus (Figure 1).

### 3.4. Average Nucleotide Identity (ANI) Values and Genomic Guanine–Cytosine Content (DNA G+C Content) 

ANI is considered the optimal method for determining relatedness between bacterial species, replacing DDH as the gold standard methodology [20]. ANI scores below 95% denote divergence and are analogous to 70% DDH. The maximum ANI score generated in this study was 81.65% with *Cedecea neteri* (ATCC 33855), supporting the assertion that strain ZA_0188^T^ is a novel species (Table 1). The DNA G+C content of strain ZA_0188^T^ was determined as 53.0 mol%, >1% lower than previously reported in the *Cedecea* genus (Table 2).

### 3.5. Morphological Characterisation 

**Figure 2:**
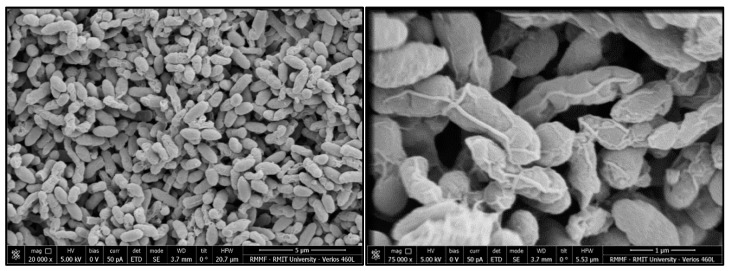
Scanning electron micrographs of strain ZA_0188^T^ showing cell morphology. Bars 5 µm (**left**) and 1 µm (**right**).

Cell morphology, as examined by SEM (Figure 2), indicated cells were rod-shaped and approximately 0.5 µm by 2 µm in size.

### 3.6. Phenotypic Characterisation

The biochemical characteristics of strain ZA_0188^T^ are summarised in Table 2. Strain ZA_0188^T^ was distinct from other *Cedecea* species such as colistin- and cephalothin-sensitive, lipase-, sorbitol-, sucrose-, and Voges-Proskauer-negative, and melibiose-, arabinose-, arginine-, and rhamnose-positive. 

Preliminary tests suggest strain ZA_0188^T^ degrades cellulose shown by growth on solid media with carboxymethyl cellulose and exhibits high level tolerance to eucalyptus oil compared to *Escherichia coli* and *Klebsiella pneumoniae*, with Lowest Observable Effect Concentrations (LOEC) of 3%, 1.5% and 1.5%, respectively. Given that strain ZA_0188^T^ was isolated from koala, these results have led us to speculate about the role this organism plays in the degradation of recalcitrant dietary compounds in the koala hindgut. 

### 3.7. MALDI-TOF Biotyper Analysis

The Bruker Biotyper enables rapid identification of bacterial species [24]. Strain ZA_0188^T^ could not be identified using this method, with similarity scores falling between 0.00 and 1.69. As *C. davisae* is present in the Bruker database, this result supports the assertion that strain ZA_0188^T^ represents a novel species. Mass spectrum (4000–10,000 Da range) data from strain ZA_0188^T^ have been uploaded to the local reference library for future identification.

## 4. Conclusions

Polyphasic taxonomic analysis suggested that strain ZA_0188^T^ represents a novel species within the genus *Cedecea*, for which the name *Cedecea colo* sp. nov. is proposed.

### 4.1. Description of Cedecea colo Species Novel

Cells are Gram-negative, rod-shaped, 0.5 µm in width and 2 µm in length, after 3 days incubation on NA in an aerobic environment at 37 °C. Colonies are irregular, umbonate, 2 mm in diameter and slightly pink on NA. Strong growth is observed on NA at 25 °C and 37 °C under aerobic conditions. Cells are motile; arginine decarboxylase, lipase, oxidase, Dnase- and Voges–Proskauer-negative; cephalothin- and Polymyxin B -sensitive; eucalyptus-oil-resistant, catalase- and cellulase-positive; utilise citrate as a sole carbon source; reduce nitrate; are rhamnose-, arabinose- and melibiose-positive; are gelatinase-negative; and are unable to ferment sucrose or sorbitol. The DNA of the organism has a G+C content of 53.0 mol (%). *Cedecea colo* (*colo:* Gundungurra people’s word for koala). The GenBank accession numbers of the 16S rRNA and draft genome sequence of type strain are SUB6872305 and SOYS00000000, respectively.

### 4.2. Importance of Cedecea colo sp. nov.

The isolation of *Cedecea colo* sp. nov and its whole genome sequence is starting to provide insights into hindgut microbiota of koalas, and into what contributions such microbes may be making towards the health and nutrition of koalas who may depend on them for their very survival. This may include providing protection from the extreme environment of eucalyptus oil, and other harsh products found in eucalypt leaves. Currently, the pathway leading to detoxification of eucalypt oil has not been identified, but genes involved in resistance to arsenic/arsenite are known and are present in *Cedecea colo* sp. nov, no doubt such genes can provide protection to increased levels of arsenic/arsenite that are sequestered by eucalypts.

## Figures and Tables

**Table 1:** In silico phylogenetic characterisation comparing strain ZA_0188^T^ with other Enterobacteriaceae species.

Organism	NCBI/GenBank Accession	ANI Two-Way (%)	16S rRNA Similarity (%)	GroEL Similarity (%)
*C. neteri* strain M006	NZ_CP009458.1	80.87	97.31	90.87
*C. neteri* strain SSMD04	NZ_CP009451.1	81.03	97.24	90.42
*C. neteri* ATCC 33855	NZ_BCTL01000007.1	81.65 *	97.12	90.51
*C. davisae* DSM4568	NZ_ATDT00000000.1	81.10	97.17	92.17 *
*C. freundii* ATCC 8090	NZ_ANAV00000000.1	79.34	96.97	90.47
*C. universalis* NCTC 9529	NZ_CP012257.1	79.65	96.07	88.91
*E. aerogenes* KCTC 2190	NC_015663.1	79.61	96.82	88.85
*E. cloacae* ATCC 13047	NC_014121.1	79.55	96.83	89.27
*E. xiangfangensis* LMG27195	NZ_CP017183.1	79.59	97.93 *	88.96
*E. albertii* KF1	NZ_CP007025.1	79.17	96.56	90.53
*E. coli* strain O157H7	NC_002695.1	79.15	97.11	90.24
*E. fergusonii* ATCC35469	NC_011740.1	79.22	96.76	90.30
*K. pneumonia* ATCC 13884	NZ_ACZD00000000.1	79.62	96.14	88.61
*K. oxytoca* CAV1374	NZ_CP011636.1	79.65	97.04	90.24
*L. adecarboxylata USDA -60222*	NZ_CP13990.1	79.39	97.31	88.77
*R. ornithinolytica* ATCC 31898	NZ_BCYR01000001	78.58	96.11	88.97
*S. bongori* NCTC 12419	NC_015761.1	79.08	97.38	90.12
*S. enterica serovar* Typhi strain CT18	NC_003198.1	79.55	97.79	89.62
*S. enterica servoar Typh.* ATCC 13311	CP009102.1	79.70	97.86	89.62
*S. plymuthica* NBRC 102599	BCTU00000000.1	78.39	95.26	90.89
*S. flexneri* ATCC 12022	NZ_JPPN00000000.1	79.15	96.53	90.06
*S. sonnei* 53G	NC_016822.1	79.30	97.38	90.06

* These values are the highest in their respective columns.

**Table 2:** Comparison of phenotypic characteristics of strain ZA_0188^T^ and closely related Enterobacteriaceae species. **Taxa: 1:** Strain ZA_0188; **2:** *E. coli*; **3:** *Cedecea neteri*; **4:** *Citrobacter freundii*; **5:** *Enterobacter cloacae*; **6:** *Enterobacter aerogenes*; **7:** *Klebsiella pneumoniae*; **8:** *Klebsiella oxytoca*; **9:** *Leclercia adecarboxylata*; **10:** *Salmonella bongori*; **11:** *Salmonella enterica* subsp. arizonae; **12:** *Salmonella enterica* serovar Typhi; **13:** *Serratia plymuthica*; **14:** *Shigella flexneri*; **15:** *Yersinia enterocolitica*. **+**: positive result; **(+)**: weak positive result (below 20%); **-** : negative result. Reference taxa data were taken from Farmer (1999).

Characteristic	1	2	3	4	5	6	7	8	9	10	11	12	13	14	15
DNase	-	-	-	-	-	-	-	-	-	-	(+)	-	+	+	(+)
Oxidase	-	-	-	-	-	-	-	-	-	-	-	-	-	-	-
Catalase	+	+	+	+	+	+	+	+	+	+	+	+	+	+	+
Lipase	-	-	+	-	-	-	-	-	-	-	-	-	+	+	+
*Hydrolysis of:*															
ONPG	+	+	+	+	+	+	+	+	+	+	+	-	+	(+)	+
Arginine	-	-	+	+	+	-	-	-	-	+	+	(+)	-	(+)	-
*Decarboxylation of:*															
Lysine	-	+	-	-	-	+	+	+	-	+	+	+	-	-	-
Ornithine	-	+	-	-	+	+	-	+	-	+	+	-	-	-	+
Citrate Utilisation	+	-	+	+	+	+	+	+	-	+	+	-	+	-	-
H_2_S Production	-	-	-	+	-	-	-	-	-	+	+	+	-	-	-
Urease	-	-	-	+	+	(+)	+	+	+	-	-	-	-	-	+
Indole Test	-	+	-	+	-	-	-	+	+	-	(+)	-	-	+	+
Voges-Proskauer Test	-	-	+	-	+	+	+	+	-	-	-	-	+	-	(+)
Gelatinase	-	-	-	-	-	-	-	-	-	-	-	-	+	-	-
*Fermentation of:*															
Glucose	+	+	+	+	+	+	+	+	+	+	+	+	+	+	+
Mannose	+	+	+	+	+	+	+	+	+	+	+	+	+	+	+
Inositol	-	-	-	-	+	+	+	+	-	-	-	-	+	-	+
Sorbitol	-	+	+	+	(+)	+	+	+	-	+	+	+	+	+	+
Rhamnose	+	+	-	+	+	+	+	+	+	+	+	-	-	(+)	(+)
Sucrose	-	+	+	+	+	+	+	+	+	-	(+)	-	+	(+)	+
Melibiose	+	+	-	+	+	+	+	+	+	+	+	+	+	+	(+)
Arabinose	+	+	-	+	+	+	+	+	+	+	+	(+)	+	+	+
Nitrate reduction	+	+	+	+	+	+	+	+	+	+	+	+	+	+	+
DNA G+C (mol %)	53	48–52	54–55	50–51	52–54	54–56	56–58	55–58	52–55	51–52	50–53	50–53	53–57	49–51	47–50
